# Supplementary material for: Protective role of resolvin D1, a pro-resolving lipid mediator, in nonsteroidal anti-inflammatory drug-induced small intestinal damage
Source: PLoS One. 2021 May 4;16(5):e0250862. doi: 10.1371/journal.pone.0250862 (PMC8096073; doi:10.1371/journal.pone.0250862)
Supplement: S1 Table — (DOCX) [file pone.0250862.s001.docx]

S1 Table

The PCR primers and TaqMan Probes

| *Gene* | Primers or Probes | Sequence or Product # | Source |
| --- | --- | --- | --- |
| *Il-1b* | Primer (forward) | 5'-ACAGGCTCCGAGATGAACAAC-3' | Sigma-Aldrich |
|  | Primer (reverse) | 5'-CCATTGAGGTGGAGAGCTTTC-3' | Sigma-Aldrich |
|  | Probe | 5'-FAM-GAAAAAGCCTCGTGCTGTCGGACCCATAT-TAMRA-3' | Sigma-Aldrich |
| *Cxcl1* | Primer (forward) | 5'-AGAGCTTGAAGGTGTTGCCCT-3' | Sigma-Aldrich |
|  | Primer (reverse) | 5'-CTCGCGACCATTCTTGAGTGT-3' | Sigma-Aldrich |
|  | Probe | 5'-FAM-CCCACTGCACCCAAACCGAAGTCATA-TAMRA-3' | Sigma-Aldrich |
| *Tnf-a* | Primer (forward) | 5'-TCATGCACCACCATCAAGGA-3' | Sigma-Aldrich |
|  | Primer (reverse) | 5'-GAGGCAACCTGACCACTCTCC-3' | Sigma-Aldrich |
|  | Probe | 5'-FAM-AATGGGCTTTCCGAATTCACTGGAGC-TAMRA-3' | Sigma-Aldrich |
| *12/15-Lox* | (Primers and probe set) | # Mm00507789_m1 | Thermo Fisher Scientific |
