# Supplementary figures and images for: Protective role of resolvin D1, a pro-resolving lipid mediator, in nonsteroidal anti-inflammatory drug-induced small intestinal damage
Source: PLoS One. 2021 May 4;16(5):e0250862. doi: 10.1371/journal.pone.0250862 (PMC8096073; doi:10.1371/journal.pone.0250862)

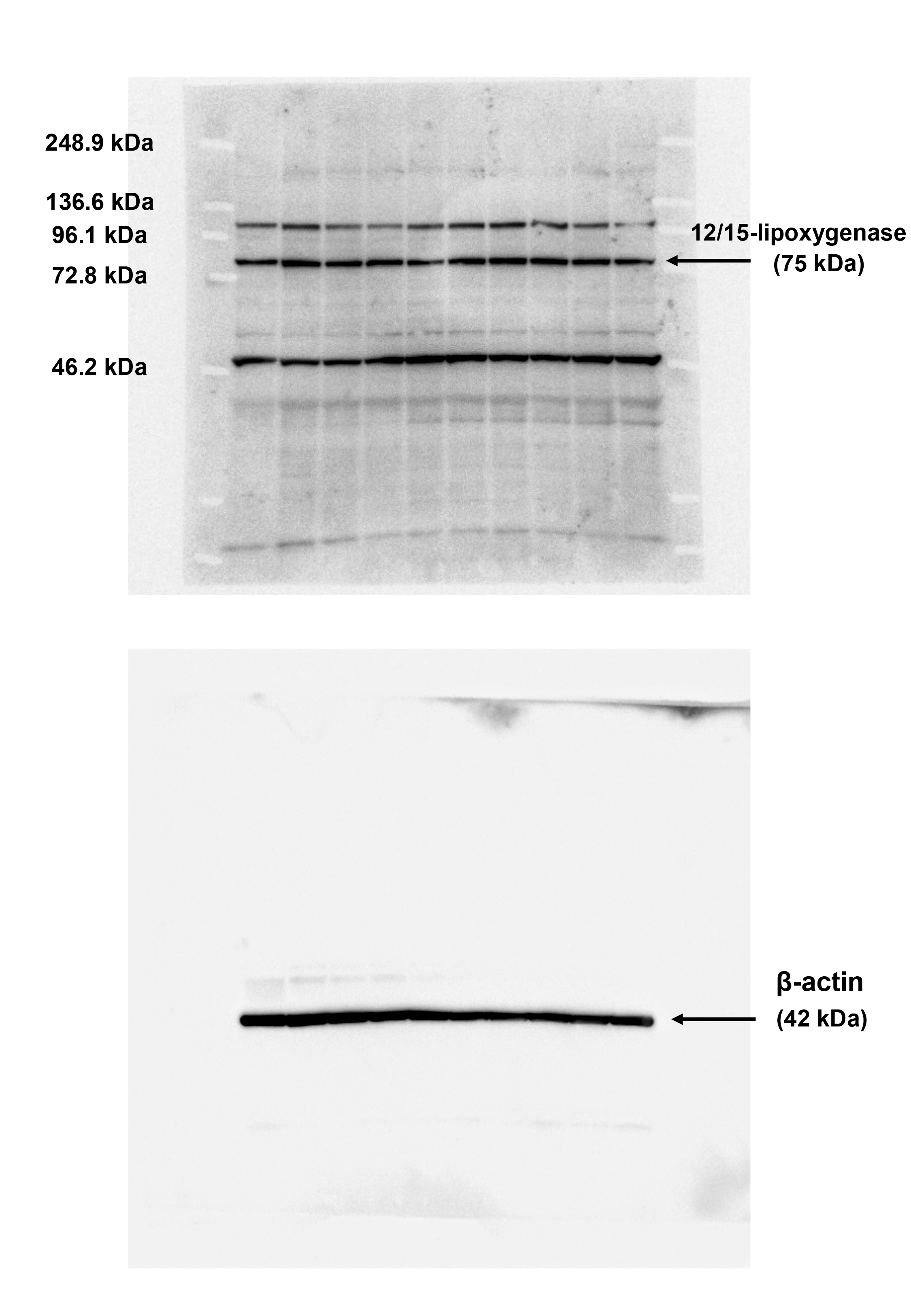

Supplement: S1 Fig — (TIF) [file pone.0250862.s002.tif]

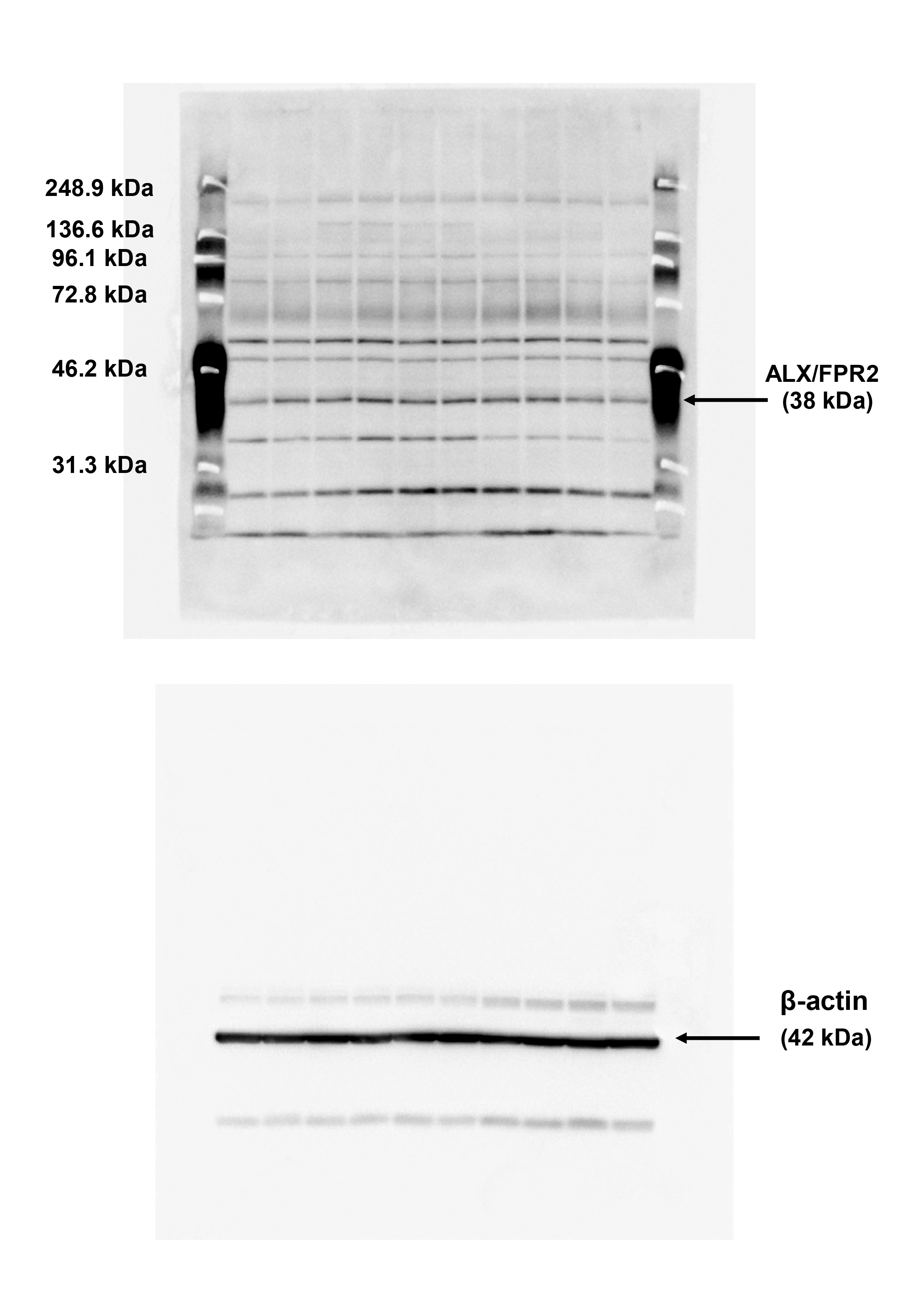

Supplement: S2 Fig — (TIF) [file pone.0250862.s003.tif]
